# The treatment of mental illness in faith-based and traditional healing centres in Ghana: perspectives of service users and healers

**DOI:** 10.1017/gmh.2020.21

**Published:** 2020-10-14

**Authors:** Jessica E. Lambert, Fred Nantogmah, Adam Yahaya Dokurugu, Hanan Alhassan, Sandow Stanislaus Azuure, Peter Badimak Yaro, Jeanette Kørner

**Affiliations:** 1 Dignity- Danish Institute Against Torture, Kobenhavn, Denmark; 2 BasicNeeds-Ghana, Accra, Ghana

**Keywords:** Ghana, prayer camps, traditional healers, trauma

## Abstract

**Background:**

The maltreatment of people with mental illness in Ghana's traditional and faith-based healing centres, including shackling, flogging, and forced fasting, has been documented by numerous sources. Such treatment is potentially traumatising and may exacerbate mental health problems. Despite widespread use, few studies have focused on experiences and characteristics of people who seek traditional healing for mental illness or healers' perspectives treatment of these conditions.

**Method:**

Purposeful sampling was used to recruit 82 individuals who were treated in healing centres and 40 traditional healers; all took part in semi-structured interviews. Those treated were asked about experiences in centres and assessed for prior trauma exposure, posttraumatic stress, and functional impairment. Healers were asked about beliefs and practices related to the treatment of mental illness.

**Results:**

Individuals treated in centres and healers generally believed that mental illness has a spiritual cause. Approximately 30.5% of those treated in centres were exposed to maltreatment; despite this, half would return. Individuals with a history of trauma were more likely to report maltreatment in the centre and had higher symptoms of posttraumatic stress. Most participants had impaired functioning. Healers who used practices like shackling believed they were necessary. Most healers were willing to collaborate with the official health structure.

**Conclusion:**

Results provide insight into the treatment of mental illness by traditional healers in Ghana and the need for trauma-informed mental health services. Findings also highlight the importance of considering cultural beliefs when attempting to implement mental health interventions in the region.

The severe maltreatment of individuals with mental illness in faith-based and traditional healing centres in Ghana has recently received attention in the international media, perhaps most notably in investigative reports by the BBC^†^^1^ and The Guardian^2^ in which individuals were pictured chained to trees and caged in prisonlike cells. Other types of potentially harmful practices have also been documented, including non-consensual admission, shackling, flogging, and forced fasting (Méndez, 2015; Human Rights Watch [HRW], 2019). These treatments constitute human rights violations and are potentially traumatising (Méndez, 2015). Nevertheless, faith-based and traditional healers continue to be the first point of contact for many people with mental illness in Ghana (Ofori-Atta *et al*., 2010), with an estimated 70% of the population turning to traditional healing for relief from their symptoms (Ae-Ngibise *et al*., 2010). Despite their widespread use, little is known about the characteristics and experiences of individuals who seek help in these centres or the healers' perspectives on the treatment of mental illness.

In Ghana, traditional and faith-based healers (TFBH) are a heterogeneous group that includes Christian and Islamic faith-healers, herbalists, and shrine priests or medicine men (Kpobi and Swartz, 2018; Badu *et al*., 2019; Kpobi *et al*., 2019). Practices of the healers vary widely. Some provide consultations. Others run residential facilities, commonly referred to as prayer camps or healing centres, where people stay for weeks or even years. Herbalists primarily prescribe tonics and ointments; faith-based healers use scriptures to guide treatment which may include prescribed behaviours, including fasting and prayer (Badu *et al*., 2019). Although Ghana's mental health law (Act 846, 2012) banned flogging, shackling, and chaining, the practice has continued at healing centres throughout the country (HRW, 2019).

The precise number of TFBH is not known because not all are regulated by the Traditional Medicine Practice Council or belong to the Ghana Federation of Faith-based and Traditional Healers. Ae-Ngibise *et al*. (2010) estimated that there is one healer for every 200 people. In contrast, the most recent data available indicated there are a total of 39 psychiatrists (Ghana Mental Health Authority, 2019), 86 clinical psychologists, and 47 counselling psychologists (Ghana Psychology Council, 2020) for the entire country. With a population nearing 30 million, it is not surprising that only 2% of individuals with mental illness receive treatment in the official healthcare system (Eaton and Ohene, 2016).

In addition to accessibility, however, the decision of where to seek help for mental illness is influenced by cultural belief systems (Badu *et al*., 2019). The meaning a person attributes to the aetiology of their symptoms has profound implications for help-seeking (Hinton and Lewis-Fernandez, 2010). The population of Ghana is one of the most religious in the world (Gallup, 2017), and symptoms of mental illness are commonly believed to be spiritual or supernatural, caused by evil spirits, punishment from God, or curses (Opare-Henaku and Utsey, 2017; Kpobi and Swartz, 2018; Salifu Yendork *et al*., 2018). As such, the problem is often seen as needing a spiritual cure (Ae-Ngibise *et al*., 2010).

An adequate understanding of the cultural context, including beliefs and attitudes towards mental illness, is essential for effectively implementing mental health and psychosocial support (MHPSS) interventions (Faregh *et al*., 2019). The purpose of this exploratory study was to understand better the treatment of mental illness in Ghana's traditional and faith-based healing centres from the perspective of people who have been treated there (i.e. service users) and healers. We were specifically interested in the degree to which individuals who were exposed to human rights violations in healing centres and had a history of potentially traumatising life events experienced posttraumatic stress symptoms. The study was part of a larger project aimed at determining the need for trauma-informed MHPSS in Ghana. TFBH were interviewed to understand their beliefs on mental illness, treatment practices, and willingness to collaborate with the official health structure.

## Method

### Study location

The study was conducted in the Ga West Municipality in the Greater Accra Region and Zabzugu District in the Northern Region. We selected these areas because of accessibility to the populations and to compare potential differences between urban and rural settings. Ga West Municipality, the more urban area, has a population of just under 270 000 (Ghana Statistical Service, 2019) in an area of approximately 305 km^2^. The predominant religions are Christianity (87.9%) and Islam (8.3%), with few Traditionalists (0.3%) (Ghana Statistical Service, 2014a). Approximately 92% of the population in this district was literate in 2010, and the most common employment was in service/sales (38%), craft/trade (22.6%) and professional or managerial (14.2%) sectors.

Zabzugu District is one of 26 districts in the Northern Region with a population of approximately 79 000 in an area of about 1100 km^2^ (Ghana Statistical Service, 2019). The most common religions are Islam (49.4%) and Traditionalist (36%) followed by denominations of Christianity (10.6%) (Ghana Statistical Service, 2014b). In 2010, 30.8% of the population was literate (World Bank, 2010). Employment (86.3%) is primarily in the agricultural sector.

### Participants

#### Service users

Eighty-two participants who had been treated for a mental health problem by a TFBH took part in the study (see Table 1). Participants in Ga West were primarily employed in trade or casual work. Those in the Zabzugu District worked primarily in farming. Most participants in both locations were living with family (*n* = 72).

**Table 1.** Demographic information for service users

	Zabzugu District *n* (%)	Ga West Municipality *n* (%)
Gender		
Male	19 (47.4%)	18 (47.4%)
Female	25 (56.8%)	20 (52.6%)
	Age	
Mean age (s.d.)	33.68 (15.09)	34.89 (14.73)
Marital status		
Single	16 (36.4%)	17 (44.7%)
Married	14 (31.8%)	10 (26.3%)
Divorced	9 (20.5%)	6 (15.8%)
Widowed	5 (11.4%)	4 (10.5%)
Missing	–	1 (2.6%)
	Children	
Mean number of children (s.d.)	1.89 (1.52)	3.93 (3.53)
Religion		
Christian	12 (27.3%)	33 (86.8%)
Islam	24 (54.5%)	3 (7.9%)
Traditional	6 (13.6%)	2 (5.3%)
Missing	2 (4.5%)	–
	Employment	
Employed	13 (29.5%)	20 (52.6%)

*Note*. Zabzugo total *N* = 44. Ga West total *N* = 38.

#### TFBH

Twenty TFBH from each location, ranging in age from 29 to 90 years (*M* = 51.75, s.d. = 14.96), took part in the study. Participants from the Zabzugu District were primarily male (*n* = 19), whereas those from Ga West Municipality were just over half female (*n* = 11). In the Zabzugu, most reported no formal education (*n* = 19), with one participant having completed primary education. In Ga, West education levels varied: five had no formal education, six completed primary school, seven completed middle school, and two had some education beyond high school. A majority (87.5%) in both locations had been in the role of healer for more than 5 years. Just over half (52.5%) of the healers inherited their function, 40% came to their role from a calling from God, and 25% through apprenticeship; some healers reported becoming a healer through more than one avenue.

### Measures

#### Service users

*Background Information***.** A semi-structured questionnaire was developed for this study to gather information on beliefs about mental illness and experiences in healing centres including the reasons for, frequency, and duration of visits to healing centres as well as exposure to potentially harmful treatments. All items included specific statements to which the respondent was asked to reply yes or no, with the option to include additional information or to refuse to answer. For example, participants were asked whether they experienced any of the following in a healing centre: chaining/shackling, denial of food, denial of water, or sleep deprivation. To avoid potential bias, we did not describe treatments as ‘potentially harmful’ during the interview. All participants were asked if they would seek treatment from a traditional healer in the future; a subset of participants was asked why or why not. We also gathered demographic information.

*Trauma Exposure*. A modified version of the Life Events Checklist for the DSM-5 (LEC-5; Weathers *et al*., 2013) was used to assess respondents' history of exposure to PTEs. The LEC is a 16-item checklist commonly used in research and clinical practice. We modified the list to be relevant to the present context by excluding some items (e.g. exposure to toxic substances). Participants were read a list of 10 PTEs, including natural disaster, fire, vehicular accident, assault by a family member, among others, and asked to respond yes or no based on whether they had experienced the event. In this study, we only assessed personal exposure to events, not witnessing or hearing about events.

*Posttraumatic Stress Symptoms (PTSS)*. The Posttraumatic Stress Disorder Checklist for the DSM-5 (PCL-5; Blevins *et al*., 2015) is a 20-item checklist used to assess symptoms of PTSS, as defined by DSM-5. Respondents were read a list of symptoms and asked to indicate how much the symptom bothered them over the past month on a scale ranging from 0 (*not at all*) to 5 (*extremely*). The PCL-5 was administered only to individuals who reported exposure to a PTE or potentially harmful treatment in a healing centre. Cronbach's alpha was 0.95 in this study.

*Functional Impairment*. The 12-item World Health Organization Disability Assessment Schedule 2.0 (WHO-DAS 2.0; WHO, 2010) was used to assess the functioning. Respondents were read statements and asked to rate the difficulty of each task during the last 30 days on a scale ranging from 0 (*none*) to 4 (*extreme*). The measure can be scored by summing items or calculating an estimated percentage the person is disabled by dividing their sum score by the maximum possible on the scale, 48. The WHO-DAS 2.0 has demonstrated cross-cultural validity for assessing general functioning among diverse populations (Federici *et al*., 2017).

#### TFBH

A semi-structured questionnaire was developed for this study to obtain information on centres from the perspective of healers. Respondents were asked to provide demographic information, general information about the centre (religious affiliation, years in existence), their perspectives on why people come to the centres, and types of treatment methods that are typically used and whether they were effective. For example, the enumerator read off a list of treatments (e.g. fasting, flogging, use of restraints) and asked the TFBH to indicate whether it was implemented in their centre. For each affirmative response, they were asked if the treatment was helpful for symptoms of mental illness (yes/no) and how. Respondents were also asked about referrals to medical centres and perspectives on collaboration with mental health professionals.

### Procedures

The Institutional Review Board of the University for Developmental Studies in Ghana approved the study before data collection began. Enumerators (*n* = 7) were university students and employees of a community service organisation with prior research training and experience. Training for this study was conducted over a 2-day workshop. Given the service users are potentially vulnerable, enumerators were trained to evaluate capacity to provide consent. Five enumerators were deployed in the sparsely populated Zabzugu District, and two were in the densely populated Ga West Municipality. Data collection proceeded in two phases. In the first phase, data were collected from service users (*n* = 51) and TFBH (*n* = 40). These data were analysed and presented during a dissemination workshop in March 2019. Based on the feedback, the second round of data collection was initiated, wherein data were collected from additional service users (*n* = 31). The protocol was the same except for one item; participants were asked why they would or would not seek treatment from a TFBH in the future.

Ghana is a multilingual country; interviews were conducted in Dagbanli, Kokomba, Twi, Ga, and English. In Accra the interviews were primarily in English or Twi; in Zabzugu they were primarily in English or Dagbanli. Our local research team primarily read in English. Before data collection, the research team agreed appropriate translation of study questions using a consensus approach (Abubakar and van de Vijver, 2017). For example, *stressful experience* was translated as *ahohiahiamu* in Twi and *ningben miisim* in Dagbanli; *angry outburst* was translated as *abufushiw* and *taabu*, in the same languages, respectively.

#### Service users

Service users were recruited through peer support groups for people with mental health problems. Inclusion criteria were (a) having been treated by a TFBH, (b) being at least 18 years of age, and (c) capable of providing informed consent. Participants were given the opportunity to ask questions after being read the consent form and were asked specific follow-up questions to ensure they understood. Each respondent was interviewed individually, in their homes or in another place where privacy could be assured; all questions were read aloud. Responses were recorded on paper forms and later entered in an electronic database. This part of the study was primarily quantitative, with a few supplementary short answer questions. Interviews lasted 60–85 min.

#### TFBH

Healers were recruited through contacts with key informants. Potential participants were invited to take part in a study on TFBH perspectives on mental illness and its treatment. All provided informed consent to take part. Interviews lasted 50–60 min.

### Data analysis

Data for the study on service users were primarily quantitative, supplemented with one open question; descriptive data were generated, together with chi-square and *t* tests to compare sub-samples. Data from TFBH included descriptive information and qualitative responses to open questions. Data from open questions were subjected to a content analysis by two members of the research team to identify key themes. Specifically, using a conventional content analysis approach outlined by Hsieh and Shannon (2005), responses to each open question were read and categories developed inductively. Because responses were relatively brief and focused, category developed proceeded in a straightforward manner. For example, in response to the question ‘Would you return to the healing centre; why or why not?’, all participants noted the degree of benefit from treatments, affordability, and/or level of satisfaction with treatment. The two researcher team members compared responses and negotiated any differences by going back to the data. The developed categories were then reviewed by a third member of the team to verify conclusions. The third member agreed with the categories as developed by the two other members and did not recommend changes.

## Results

### Treatment experiences

Most respondents from both study locations had more than one visit to a TFBH (see Table 2). The primary reasons for seeking help were mental illness (*n* = 52), spiritual concerns (*n* = 10), physical problems (*n* = 10), and epilepsy (*n* = 6). Of the people who sought help for mental illness, two stated it was caused by substance abuse and two by accidents; the remainder stated it had a spiritual or supernatural cause. Physical illnesses were attributed to infections (*n* = 3), accidents (*n* = 1), spiritual (*n* = 4) or unknown causes (*n* = 3). Four of the six people who were treated for epilepsy reported it had a spiritual cause, one an accident and one from substance abuse. Most respondents reported family members (60.7%) decided for them to seek treatment from a TFBH. In Zabzugu, 18 participants stated they continued to have a problem after treatment; of these, 13 sought help in the formal healthcare system. In Accra, 14 continued to have a problem, and 13 sought help in the healthcare system.

**Table 2.** Frequency of treatment and number of participants who reported potentially harmful treatment in healing centres

	Zabzugu District *n* (%)	Ga West Municipality *n* (%)
Number of visits		
One	5 (11.4%)	9 (23.7%)
Two to three	7 (15.9%)	10 (26.4%)
Four or more	34 (72.7%)	18 (47.4%)
Length of admission		
Less than 1 week	1 (2.3%)	7 (18.-4%)
1–4 weeks	5 (11.4%)	10 (26.5%)
2–4 months	11 (25%)	6 (15.8%)
5 months–1 year	8 (18.2%)	3 (7.9%)
More than 1 year	8 (18.2%)	0 (0%)
Not admitted	0 (0%)	12 (31.6%)
Potentially harmful treatments		
Restraints	8 (15.8%)	6 (18.2%)
Denied food	9 (20.5%)	5 (13.2%)
Denied water	10 (22.7%)	2 (5.3%)
Sleep deprived	8 (18.2%)	1 (2.6%)
No sleeping place	3 (6.8%)	3 (7.9%)
Flogging	4 (9.1%)	2 (5.3%)

*Note*. Zabzugu District total *N* = 44, 15 exposed to potentially harmful treatments. Ga West total *N* = 38, 10 exposed to potentially harmful treatments.

Twenty-five of the 82 (30.5%) respondents indicated they experienced at least one potentially harmful treatment in a healing centre (see Table 2). Most (72%) reported four or more visits to healing centres. All participants were asked if they were likely to seek treatment in a healing centre in the future. In the Zabzugu, 61.4% would return; in Ga West, only 23.7% would. Thirteen of the 25 respondents (52%) who reported some form of maltreatment stated they would likely return in the future. The majority (*n* = 10) resided in the Zabzugu.

A subsample (*n* = 31) of respondents was asked why they would or would not return. Of the 15 respondents who said they would, most indicated the treatment was beneficial (*n* = 11) and affordable (*n* = 3). One stated, ‘Because the treatment cost less than the hospital, and I will get well there’, and another ‘Because the pastor took good care of me and I am getting better.’ Seven of these individuals reported some form of potentially harmful treatment. Of the 17 who reported they would not return, most stated it was because the treatment was insufficient (*n* = 11), or they were mistreated (*n* = 5). One noted, ‘Prayer camp solved the spiritual problem, but the depression did not go away; therefore, I will not go back.’ Two of the participants who felt mistreated stated ‘I was shabbily treated. I was seen as an outcast, and my condition did not improve’ and ‘The treatment process is painful, and I had no right to question what medicine or herb is [used].’

### Trauma exposure, PTSS, and functional impairment

Of the 82 participants, 32 (39%) reported lifetime exposure PTEs, with a higher number residing in the Zabzugu District (*n* = 20). In Zabzugu, the most commonly reported event was a physical assault by a non-family member (*n* = 9) or family member (*n* = 8) and traumatic death of a family member (*n* = 8). In Ga West, the most common events were traumatic death of a family member (*n* = 4) and motor vehicle accident (*n* = 3). Participants who were exposed to potentially harmful treatments in the centres were two times more likely to have experienced a PTE at some point in their lives than participants who were not [χ^2^ (1, *N* = 82) = 18.33, *p* < 0.001].

Respondents who reported PTE or potentially harmful treatment in the healing centre were assessed for PTSS. The average score for this subsample (*N* = 40) was 31.63 (s.d. = 21.27); 22 participants had scores above the clinical cut-off of 33. Scores were not significantly different among respondents from Ga West Municipality (*M* = 25.82, s.d. = 18.91, *n* = 17) and Zabzugu District (*M* = 35.91, s.d. = 22.29, *n* = 23) [*t*(39) = 1.51, *p* = 0.14]. Of the 25 participants who reported exposure to some form of potentially harmful treatment in the centres, those who also had a history of exposure to at least one PTE had significantly higher scores on the PCL-5 (*M* = 29.80, s.d. = 27.52, *n* = 17) than those with no history of PTEs (*M* = 44.71, s.d. = 16.3, *n* = 8) [*t*(25) = −2.23, *p* = 0.035].

Approximately 72% of the sample had disability scores at or above 50% on the 12-item WHO-DAS 2.0; a score of 0% indicates no disability, whereas a score of 100% represents full disability. Average scores were similar in samples from both study locations; 65.96% in the North and 56.63% in Accra.

#### Healers

Descriptive information for the centres is shown in Table 3. In Greater Accra, 90% of the centres placed people on admission; 85% of the centres in the Zabzugu District placed people on admission.

**Table 3.** Characteristics of Healing Centres by Location

	Zabzugu District *n* (%)	Ga West Municipality *n* (%)
Type of centre		
Prayer camp	6 (30%)	11 (55%)
Traditional	13 (65%)	8 (40%)
Other	1 (5%)	1 (5%)
Religious Affiliation		
Christian	2 (10%)	10 (50%)
Islam	5 (25%)	–
Traditional	8 (40%)	2 (10%)
Not affiliated	5 (25%)	8 (40%)
Duration of Existence		
1–5 years	2 (10%)	5 (25%)
6–10 years	4 (20%)	8 (40%)
11–15 years	3 (15%)	2 (10%)
More than 16 years	11 (55%)	5 (25%)

*Note*. *N* = 20 in each location.

Respondents in both Ga West Municipality (85%) and the Zabzugu District (95%) reported that mental illness was one of the common problems treated. Mostly, 90% in Ga West Municipality and 80% in the Zabzugu District, reported spiritual or supernatural causes caused it. In Ga West Municipality and the Zabzugu District, 75 and 85% of respondents indicated, respectively, that most of the time, someone other than a service user, typically a family member, decides to seek treatment at the centre.

Respondents across both locations reported commonly using herbs (90%) and prayer (62.5%), followed by ritual sacrifice (42.5%) and use of charms (37.5%) during treatment. The most frequently used potentially harmful treatment, reported by 55% of the total sample of healers, was the use of physical restraint, which was typically done with chains or ropes. This was more common in the Ga West Municipality (*n* = 14) than in Zabzugu District (*n* = 8). Most TFBH (*n* = 12) reported restraints helped calm aggressive individuals, prevented injury, and controlled movement so medicine could be applied without interruption. One healer stated restrains ‘… makes them humble and ensures that they are not able to move away from the prayers’ whereas another stated, ‘placing them in chains helps restrain them from escaping.’

Additional potentially harmful treatment included mandatory fasting (40%), all-night vigils (30%), exorcism (22.5%), and exposure to the elements (12.5%). One healer stated: ‘Fasting makes the evil spirit to leave the body’ and another noted: ‘At times exposing clients to the elements help to calm them down for effective treatment.’ No patterns in the use of these treatments emerged based on location or type of centre.

Regarding referral, 65% of healers in the Zabzugu District and 80% of healers from Ga West Municipality reported having referred users with mental illness to the hospital. Analysis of the reasons given by respondents on why they refer users to hospitals delineated two themes. The first was that treatment at the hospitals would complement their approach, for example, by running laboratory tests for diagnosis (*n* = 7). Second, some healers (*n* = 10) acknowledged the expertise of the formal health system in statements indicating they ‘suffered from a condition that I was not able to help with’ and ‘the person could be more effectively treated at a hospital.’

Most respondents (85% from Zabzugu District and 95% from Greater Accra) agreed that collaboration with health professionals could benefit individuals with mental illness. One healer's statement reflects a common theme: ‘Treatment or good health is both spiritual (which we do here) and physical wellbeing (we leave for the hospital).’ Of the three respondents who did not believe collaboration would be beneficial, only one provided a rationale; this individual stated ‘The hospital will take the glory alone leaving the healer out. They will say the patient is well because of hospital treatment alone.’

## Discussion

In this study, we sought a better understanding of the treatment of mental illness by TFBH in Ghana from the perspective of individuals treated in the centres and healers. We were particularly interested in the degree to which people were exposed to potentially harmful treatments in the centres or other PTEs experienced symptoms of PTSS in order to inform the MHPSS services. Data were collected in one rural and one urban location to highlight any potential differences. Results are informative for the implementation of MHPSS interventions in the region by highlighting beliefs about mental illness, experiences at healing centres, as well as beliefs and practices of TFBH.

Although there was some variation, most service users and TFBH from both study locations believed mental illness has a spiritual or supernatural cause. This finding is consistent with previous research in Ghana (Opare-Henaku and Utsey, 2017; Kpobi and Swartz, 2018; Salifu Yendork *et al*., 2018) and potentially explains, at least in part, why people seek traditional healing, even when it involves potentially harmful practices. This has important implications, given that beliefs and attitudes affect help-seeking, engagement, and outcome of treatment (Benish *et al*., 2011; Faregh *et al*., 2019).

Limited availability of MHPSS is another potential reason people sought treatment at healing centres and could explain why a higher percentage of participants from the rural areas reported they would return, and fewer healers in the rural area made referrals to the medical system. It is also essential to recognise, however, that some people seek treatment from TFBH because they benefit (Ae-Ngibise *et al*., 2010) or have been dissatisfied with biomedical interventions (Read, 2012). Read (2012) found that some individuals who had been prescribed anti-psychotic medication discontinued the treatment because of side effects and sought spiritual healing instead. Some participants in our study described their experience in the centre as comforting and healing.

A relatively high percentage of participants in this study had been exposed to PTEs, particularly in the rural area. This finding is consistent with other research in Ghana showing high exposure to domestic violence (Adjah and Agbemafle, 2016), road traffic accidents (National Road Safety Commission, 2016), and other PTEs (Ben-Ezra *et al*., 2020). Although research on the impacts of PTEs in Ghana is scant, a recent study by Ben-Ezra *et al*. (2020) showed a probable prevalence of 17% for PTSD among university-educated adults. In our study, over half of individuals exposed to PTEs had elevated symptoms. Individuals with a history of PTEs were more likely to report potentially harmful treatment in the centres. Participants with both prior trauma and maltreatment in the centres had the most severe PTSS. One potential explanation is that people who present with symptoms of PTSS (e.g. flashbacks, arousal) are more likely to encounter certain types of treatments in the centres, but additional research is needed to confirm this proposition. Our findings together with those from Ben-Ezra *et al*. (2020) suggest there is a need for trauma-informed MHPSS in this context.

In recent years there has been an increased focus on scaling MHPSS interventions in low- resource countries. It is essential to tailor such interventions to be appropriate for the context, bearing in mind the local belief systems and practices (Faregh *et al*., 2019). One way of proceeding is to collaborate with TFBH (Arias *et al*., 2016). There is a long history of collaboration between TFBH and the formal health structure in Ghana. Ghana Mental Health Authority, for example, published guidelines on mental health treatment for TFBH (GMHA, 2018) and has implemented programmes to facilitate collaboration between healers and community health workers. Our study and others (Arias *et al*., 2016) suggest that many TFBH are willing to work together with mental health professionals.

When considering collaboration, however, it is also important to consider ethical implications. Over half of the TFBH in this study admitted to using forced restraint even though it was banned in 2012. Such practices may be difficult to change. In a randomised controlled trial where the experimental group was treated with medications administered by psychiatric nurses while simultaneously being treated by TFBH, the number of days spent in chains was not reduced despite improvement in symptoms (Ofori-Atta *et al*., 2018). Most healers who used physical restraint and other potentially harmful treatments in our study believed these practices were necessary for and beneficial to the healing process. Further investigation into why these practices continue and effective ways of changing them is needed.

Results must be interpreted considering the limitations. The sample size was relatively small, which limits the degree to which we can draw conclusions about subsamples. We only asked participants if they had been treated in one or more types of TFBH centres, thus were not able to draw conclusions on the frequency or types of treatments at different types of centres. We did not ask specific questions about experiences with biomedical treatments. We were not able to detect gender differences from the data we gathered; future research might explore potential differences for males and females.

We used purposive sampling, which may have resulted in a biased sample. Nearly all healers who took part in this study were willing to collaborate with official health structures. It is possible that those who were willing to be interviewed also hold a more positive view of the official system than other healers. Furthermore, most service users we interviewed were receiving supportive services at local mental health organisations. Thus, findings may not be representative of the larger populations. We did not collect data on the specific centres where service users sought treatment; thus, it is not possible to determine if they received treatment at the same centres as other participants or TFBH.

Although the research team agreed on the appropriate translation of items before data collection, it is possible there were inconsistencies that are impossible to evaluate. Future research is needed to understand better if a Western conceptualisation of PTSD captures the experience of trauma-exposed individuals in Ghana.

Despite the limitations, this study yielded information to inform the implementation of MHPSS. Findings highlight the importance of considering culturally based beliefs and practices related to mental illness in Ghana, as well as the relevance of trauma-informed work in this region. It may be beneficial to provide training for TFBH on the impacts of PTEs and other types of adversity. Research is needed to identify best practices in collaboration between biomedical and traditional healing as well as strategies for implementing MHPSS in ways that are respectful of the culture and assures effective, ethical treatment for those in need.

## Footnotes

† The notes appear after the main text.

1 https://www.bbc.com/news/av/world-africa-45800263/ghana-prayer-camps-chain-residents-with-mental-health-problems

2 https://www.theguardian.com/global-development/2020/feb/03/all-we-can-offer-is-the-chain-the-scandal-of-ghanas-shackled-sick
